# *In vitro* co-culture system for investigating Armillaria root rot in *Prunus* spp. using a fiber-supported liquid approach

**DOI:** 10.1371/journal.pone.0310314

**Published:** 2024-09-23

**Authors:** Alejandro Calle, Jeffrey Adelberg, Guido Schnabel, Jacqueline Naylor-Adelberg, Jhulia Gelain, Yeter Karakoc, Jared Weaver, Christopher Saski, Ksenija Gasic

**Affiliations:** 1 Department of Plant and Environmental Sciences, Clemson University, Clemson, SC, United States of America; 2 Fruit Production Program, Institut de Recerca i Tecnologia Agroalimentàries (IRTA), Lleida, Spain; Tocklai Tea Research Institute, INDIA

## Abstract

*In vitro* co-culture techniques that allow the growth of plants and pathogens under controlled environmental conditions are being used to re-create host plant infection. These approaches reduce infection times, promote reproducibility, and enable a rapid evaluation of plant-pathogen interactions. As a result, these systems have become essential in breeding programs aimed at developing plant resistance to diseases. In this study, we developed and validated an *in vitro* co-culture system to investigate the Armillaria root rot (ARR) affecting *Prunus* spp. This disease, caused by fungi *Armillaria* spp. and *Desarmillaria caespitosa*, poses a severe threat to the stone and nut fruit industry due to the susceptibility of most commercial rootstocks to infection and the lack of effective management options for its control. The system consists of a fiber-supported liquid approach in sterile plastic vessels that allows a fast and reproducible fungal infection under controlled environmental conditions. The floor of the vessels was covered with a polyester-fiber matte and a germination paper that served as an interface between the mycelia and the plant roots. The vessels were subjected to inoculation with *Armillaria mellea* and *D*. *caespitosa*, and three *Prunus* genotypes (‘Guardian^®^’, ‘MP-29’, and *Prunus cerasifera* ‘14–4’) were co-cultured with both fungi. Disease progression and plant and fungal biomass were monitored during co-culture. The presented *in vitro* co-culture approach facilitates the concurrent growth of *Armillaria/Desarmillaria* spp. and *Prunus* spp., excluding most of the limitations associated with greenhouses and field experiments. This system provides consistent and reproducible conditions for investigating a prominent plant disease affecting *Prunus* spp.

## 1. Introduction

Enhancing disease resistance/tolerance in plants has become a fundamental goal of modern agriculture. To achieve this, a symbiotic association between plant breeders and pathologists is needed to establish the breeding preferences and techniques [1]. Traditionally, methodologies for exploring the plant-pathogen interaction required field and greenhouse experiments to gain essential insights for subsequent resistance selection [2, 3]. Nevertheless, these approaches pose several challenges. Field screenings require replication and multi-year evaluations and are prone to environmental fluctuations that can impact inoculum levels, resulting in high rates of unsuccessful inoculations [4–6]. These drawbacks made *in vitro* techniques essential for studying the plant-pathogen interaction and to improve traditional resistance breeding systems [7]. *In vitro* systems control environmental conditions such as light, humidity, or temperature and restrict confounding biotic factors, allowing researchers to investigate plant-pathogen interactions under specific conditions [8, 9]. By eliminating the effects of environmental variability, *in vitro* co-culture systems provide more consistent results, replication, and comparison. Additionally, *in vitro* systems offer a safe and controlled environment for the plant pathogen, which minimizes contamination risks and disease spread to the environment [10]. Thus, *in vitro* approaches allow a rapid evaluation of many plants, reducing the time and resources required for field evaluations, making them valuable tools for studying plant responses to biotic and abiotic stresses [11].

Armillaria root rot (ARR), a root-borne disease caused by *Armillaria/Desarmillaria* pathogenic fungi, is considered a primary threat to plant health, particularly for forest and stone fruit trees [12]. The fungi causing this disease can persist for extended periods in a diverse range of hosts [13], resulting in the destruction of woody roots and the collapse of infected trees during the prime production phase [14]. In the US, three primary fungi [*Armillaria mellea* (Vahl) P. Kumm, *Desarmillaria caespitosa* (Scop.) R. A. Koch & Aime (= *D*. *tabescens*), and *Armillaria soildipes* Peck (= *A*. *ostoyae*)] have been identified as impacting stone and nut fruits (*Prunus* species) [15]. However, despite the importance of this disease and its economic impact [16], no effective management options are available, with most of the *Prunus* rootstocks susceptible to infection [17, 18]. In peach orchards, the only viable option to manage the disease is a cultural approach that involves soil removal to expose the root crown, which has been demonstrated to delay disease onset by up to two years [19, 20]. Thus, developing horticulturally acceptable rootstocks resistant to ARR was identified as the only long-term solution to enhance the economic viability of the stone and nut industries affected by the disease [21].

Initial research on ARR was carried out on *Armillaria/Desarmillaria* hotspots [2–4, 22]. Although these field trials provided valuable insights into plant-pathogen interaction, prolonged periods for infection assessment were required. Similarly, greenhouse assays involving inoculating plants with *Armillaria/Desarmillaria* species exhibited elevated frequencies of unsuccessful inoculation and delayed infection rates [23, 24]. Thus, *in vitro* co-culture systems involving *Armillaria/Desarmillaria* and *Prunus* species were recently developed to overcome most of these challenges [8, 25, 26]. Baumgartner et al. [25] were the first to develop an *in vitro* tube-based assay to screen for ARR-resistant almond rootstocks. In this protocol, *Prunus* genotypes were cultured in an agar-based medium and subsequently inoculated with *A*. *mellea* and *D*. *caespitosa* for two months. The degree of resistance to infection was then determined based on the visual observation of plant mortality and survival. Similarly, Adelberg et al. [8] modified this *in vitro* co-culture protocol by replacing the semi-solid agar-based medium with a phenolic foam. This modification improved aeration, reduced variation in inoculum pressure, and allowed for the successful rooting of peach genotypes that were previously difficult to root in the agar-based media. Another rapid and holistic approach for ARR screening in *Prunus* species was also developed by Devkota and Hammerschmidt [26]. This *in vitro* system considerably reduces the screening time from years to weeks and enables the measurement of infection levels during fungal penetration. Furthermore, this assay can detect host defenses and antifungal compounds of root periderm from resistant genotypes [26, 27].

These newly developed *in vitro* assays for investigating ARR offer advantages such as reduced infection time, increased reproducibility, and the ability to screen for resistant genotypes. Nevertheless, these approaches have certain limitations. For instance, in the *in vitro* assays developed by Baumgartner et al. [25], the spatial confinement of plant and fungal growth limits the availability of plant tissues, and in most woody plant species, including those in the *Prunus* genus, roots grown in semisolid agar media tend to be thicker and lack secondary branching, which exacerbates mortality as plants age [28]. Adelberg et al. [8] improved root quality, but root and fungal tissues were not readily available for analysis over several weeks of co-culture due to degradation of root tissue by the fungus and the affected tissue being intertwined in the foam matrix [29]. Considering these drawbacks and the increasing demand for plant/fungi-derived metabolites or transcriptomes, which require a large amount of tissue for analysis, underscores the need to improve *in vitro* co-culture assays. Therefore, this study introduces an enhanced *in vitro* co-culture methodology for *Armillaria/Desarmillaria* and *Prunus* species, utilizing a fiber-supported liquid system [30, 31], which has been observed to produce large quantities of woody plant roots in ornamental species of *Hydrangea* and *Clematis*. Our current approach builds upon the techniques previously proposed by Baumgartner et al. [25] and Adelberg et al. [8], facilitating the growth of plants and fungi under identical conditions and allowing for the generation of substantial quantities of plant and fungi-related compounds for downstream analyses.

## 2. Material and methods

The protocol described in this peer-reviewed article is published on protocols.io dx.doi.org/10.17504/protocols.io.rm7vzj56xlx1/v1 and is included for printing purposes as S1 File.

### 2.1 Experimental design

This protocol explains the establishment of an *in vitro* co-culture system designed to facilitate a quick and easy infection of *Prunus* roots with the soil-borne fungi *Armillaria mellea* and *Desarmillaria caespitosa*. The procedure encompasses the development and optimization phases of growth systems for both organisms, the plants and fungi, in isolation and when combined. Detailed descriptions of these processes are provided below.

### 2.2 Materials

- 20 × 150 mm culture tubes (Stellar Scientific; Baltimore, MD, USA, Cat. N°: SKU:GS-1522)
- Autoclavable polypropylene culture tube closures (General Laboratory Supply; Pasadena, TX, USA, Cat. N°: T3054-4)
- Magenta™ GA-7 vessels (Merck, Darmstadt, Germany, Cat. N°: V8505-25EA)
- Petri plates (VWR International, Radnor, PA, USA, Cat. N°: 391–0579)
- Parafilm ‘M’ laboratory film (Sigma Aldrich; Darmstadt, Germany; Cat. N°: P7668)
- 15 mL Pyrex^®^ Ten Broeck tissue grinder with a pour spout (Corning, Tewksbury, MA, USA; Cat. N°: 7727–15)
- Ultra-clear porous cellophane sheet (0.1 mm thick) (Research Products International, Mount Prospect, IL, USA; Cat. N°: 1080)
- Lazy-L spreader (Merck, Darmstadt, Germany, Cat. N°: Z376779)
- 1.5 mL Eppendorf^®^ tubes (Thermo Fisher Scientific Inc, Waltham, WA, USA; Cat. N°: 0030120175)
- Rectangular vessels (110 × 297 mm; Southern Sun BioSystems, Hodges, SC, USA)
- Polyester fiber matte (BioStrate™ Felt; Cropking Inc., Lodi, OH, USA)
- Germination paper (Anchor Paper Co., St. Paul, MN, USA)
- Polyvinyl chloride (PVC) sealing film (Phytotech Laboratories, Shawnee Mission, KS, USA; Cat. N°: A003)

### 2.3 Equipment

- Laminar flow hood
- Autoclave
- Articulated rocker arm
- Fungal growth incubator
- LED light NutriLED, Hubbell Lighting, Greenville, SC, USA)

## 3. Procedure

### 3.1 Plant culture

Three *Prunus* genotypes were employed: ‘Guardian^®^’, *Prunus cerasifera* ‘14–4’, and ‘MP-29’. While ‘Guardian^®^’ is susceptible to *Armillaria/Desarmillaria* infection, *Prunus cerasifera* (‘14–4’) and ‘MP-29’ (peach × plum hybrid) exhibit varying degrees of resistance to ARR [18, 29]. The establishment of ‘MP-29’ cultures involved using dormant shoots subjected to a series of surface sterilization procedures. These procedures consisted of washing the shoots with liquid detergent, immersing in 70% ethanol for 1 min, rinsing with deionized sterile water, dipping in 10% bleach for 10 min, and rinsing with deionized water twice. Next, the shoot buds were peeled and placed in 20 × 150 mm culture tubes topped with Kimble PM caps containing 20 mL of sterile Murashige and Skoog (MS) agar media (0.8%) with the pH adjusted to 6.2 using 1N NaOH [32]. The ‘Guardian^®^’ and ‘14–4’ cultures were initiated through seed sterilization and stratification. To achieve this, the fruit exocarps were soaked in 20% bleach for 10 minutes, followed by immersion in 70% ethanol for another 10 minutes. Subsequently, seeds were extracted from the fruit in a laminar flow hood and placed in culture tubes filled with 10 mL of ‘Woody Plant Medium’ [33] with a pH of 5.7. These seeds were stratified at 4°C for ten weeks. After germination, the shoot tips were micro-propagated in Magenta GA-7 vessels (Magenta Corp., Chicago, IL, USA) with MS agar medium under aseptic conditions.

Stock plants of these genotypes were maintained in Magenta GA-7 vessels through the periodic transfer of shoot tips every five weeks onto fresh medium, with five plants per box cultured in each cycle. ‘Guardian^®^’ were sub-cultured in Magenta GA-7 vessels with 45 mL of modified Quoirin and Lepoivre media containing agar (0.8%), fructose (30 g/L), ferulic acid (1 mg/L), ascorbic acid (2 mg/L), inositol (100 mg/L), glycine (2 mg/L), thiamine (1 mg/L), nicotinic acid (1 mg/L), pyridoxine (1 mg/L), 6-benzylaminopurine (2.2 μM), indole-3-butyric acid (0.025 μM), and gibberellic acid (0.15 μM) [34] with pH adjusted to 6.2. Occasional resting cycle with 16 μM indole-3-acetic acid (resting media) was considered based on the appearance of hyper multiplication. ‘MP-29’ and ‘14–4’ were propagated in Magenta GA-7 vessels with 45 mL of 0.8% agar pH 6.2 ‘New *Prunus* Medium’ [8] with 15 g/L of fructose for five weeks. Multiplication was achieved by alternating cycles of 2.6 μM 6-benzylaminopurine and 0.2 μM gibberellic acid with a resting cycle on a medium with 16 μM indole-3-acetic acid (resting medium). All *in vitro* plants were grown in 20 μmol/s/m^2^ photosynthetic photon flux density provided by cool white fluorescent tubes with 16 h/day photoperiod and 24°C.

### 3.2 Fungi preservation and inoculum preparation

*Armillaria mellea* (SCOOi149) and *Desarmillaria caespitosa* (= *D*. *tabescens*; SCGJ2.02) isolates were obtained from symptomatic peach trees near Cooley Springs (SC, USA) and near Cherokee (SC, USA), respectively. Isolates were stored in glass vials on 2% malt extract agar medium (MEA) under sterile mineral oil (Cumberland Swan, Smyrna, TN) in glass screw-top tubes at 4°C before use. The cultures were propagated in Petri plates containing malt extract agar (MEA) composed of 3% malt extract (PhytoTech Labs, Inc, Lenexa, KS, USA), 3% glucose (Fisher Scientific International, Inc, Pittsburgh, PA, USA), 1% peptone (PhytoTech Labs, Inc, Lenexa, KS, USA), and 1.5% agar Plant TC (PhytoTech Labs, Inc, Lenexa, KS, USA). The Petri plates were sealed hermetically with Parafilm ‘M’ laboratory film (Bemis Company, Inc, Neenah, WI, USA) and maintained in the dark at a constant temperature of 20°C. The cultures were refreshed every 14 days by transferring mycelial plugs to fresh MEA plates to ensure active fungal growth.

**Fig 1:**
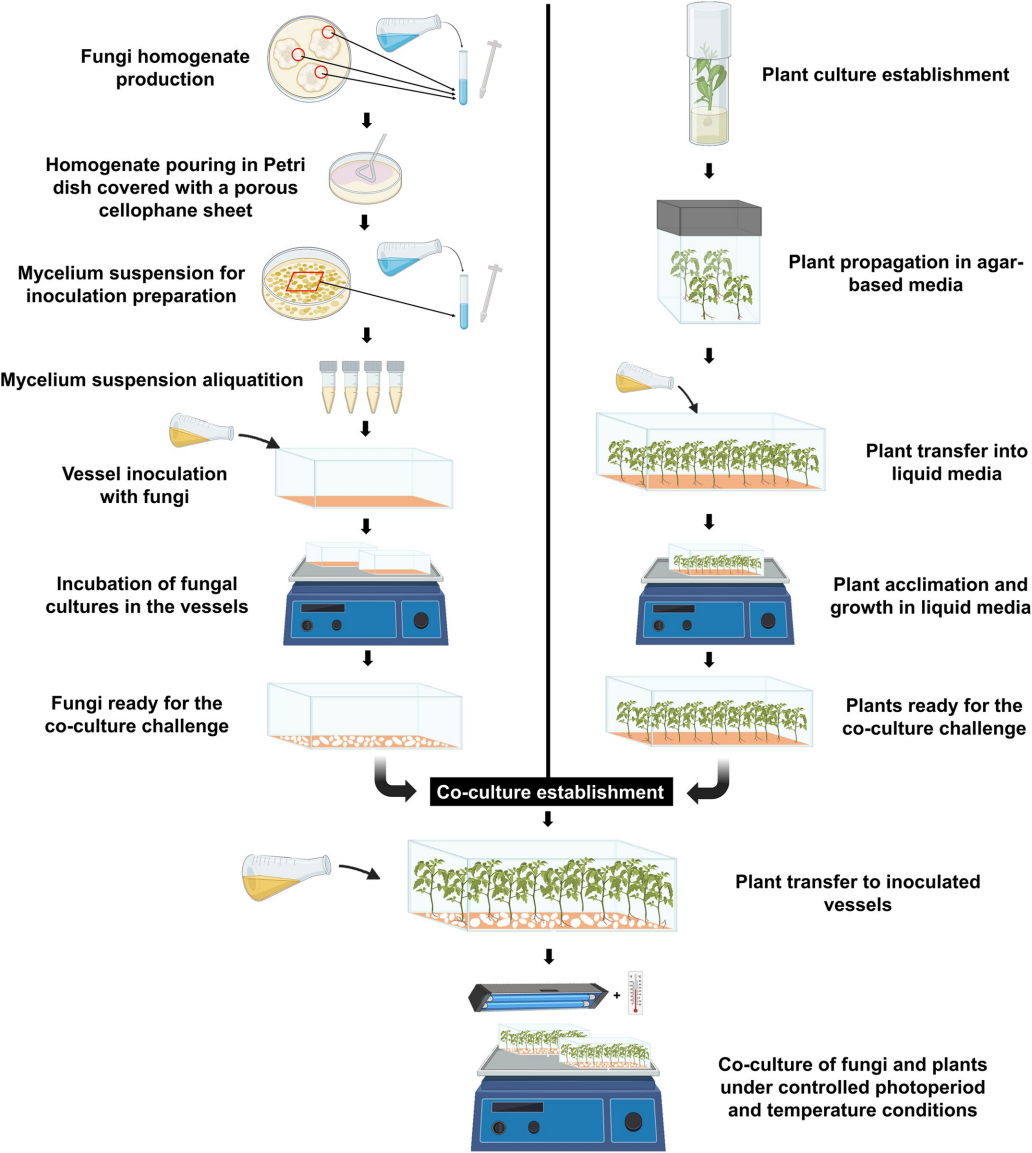
Schematic overview of fungal and plant preparation for establishing the co-culture system.

Three ten-millimeter-diameter plugs were extracted from the edge of two-week-old *Armillaria/Desarmillaria* colonies to obtain actively growing mycelium (Fig 1). Subsequently, most of the agar plug was removed, and only the aerial mycelium connected to the top half of the plug was gently homogenized with 5 mL of deionized sterile water by using a sterilized 15 mL Pyrex^®^ Ten Broeck tissue grinder equipped with a pour spout (Corning, Tewksbury, MA, USA). Then, 600 μL of this homogenate was aseptically poured onto a Petri plate that contained MEA media. Before pouring, a sterile ultra-clear porous cellophane sheet (0.1 mm thick) (Research Products International, Mount Prospect, IL, USA) was placed on top of the media to facilitate efficient and manageable harvesting. The homogenate was uniformly spread over the entire plate using a cell spreader, and the plate was incubated in the dark for 14 days after sealing. Finally, to obtain the mycelium suspension for inoculation, a 2×2 cm plug was aseptically collected from these previously mentioned plates and homogenized in 7 mL of deionized sterile water using a sterilized 15 mL Pyrex^®^ Ten Broeck tissue grinder. After that, homogenate (mycelium suspension) was aliquoted in sterile 1.5 mL Eppendorf tubes (Thermo Fisher Scientific Inc, Waltham, WA, USA) and stored at room temperature before use for no longer than an hour.

### 3.3 Co-culture (plant-fungi) establishment

The *in vitro* co-culture system employed a liquid medium supported by a polyester fiber-based paper. To set up the system, sterile plastic rectangular vessels (110 × 297 mm; Southern Sun BioSystems, Hodges, SC, USA) (S1 Fig) were autoclaved at 121°C for 20 min and cooled to room temperature (~25°C). Then, the fiber-supported paper made of a polyester fiber matte (BioStrate™ Felt; Cropking Inc., Lodi, OH, USA) was placed under the germination paper (Anchor Paper Co., St. Paul, MN, USA) [31] and set on the floor of each vessel. Afterward, 175 mL of plant growth regulator-free liquid ‘New *Prunus* Medium’ with 3% sucrose pH 5.7 was aseptically added to each vessel [8]. In preparation for the co-culture, 25 *in vitro* plants for each genotype from an agar-based medium (resting medium) were transferred to each vessel (Fig 1), which was then sealed with polyvinyl chloride (PVC) film (Phytotech Laboratories, Shawnee Mission, KS, USA). The vessels were placed on a rocker arm with an articulated shelf that provided one swing every 15 min, keeping the vessel inclined for 14 min. The plants were rooted for three weeks in vessels under cool white fluorescent tubes providing a photosynthetic photon flux density of 20 μmol/s/m2 for 16 h/day at 24°C.

Similarly, another set of rectangular Southern Sun BioSystems vessels containing 175 mL of ‘New *Prunus* Medium’, fiber matte, and germination paper were inoculated with *A*. *mellea* and *D*. *caespitosa* by pipetting 1 mL of the mycelium suspension for inoculum into each vessel (see section 3.2). Following that, vessels were sealed with PVC film and placed on an automatic rocker arm at 5 rpm under 40 μM/m^2^/s LED light 2 red 1 blue (NutriLED, Hubbell Lighting, Greenville, SC, USA) and 16 h/day photoperiod at 24°C. After ten and seventeen days of inoculation with *A*. *mellea* and *D*. *caespitosa*, respectively, co-culture was established by transferring the *in vitro* rooted plants from the liquid medium to the inoculated vessels. All inoculated vessels were supplemented with 60 mL of ‘New *Prunus* Medium’ without any plant growth regulator just before plant transferring.

### 3.4 Experimental design and disease evaluation

‘Guardian^®^’, *Prunus cerasifera* ‘14–4’, and ‘MP-29’ were challenged with *A*. *mellea* and *D*. *caespitosa* for the development and validation of this protocol. Twelve vessels were used for each genotype, with 25 plants per vessel. Five vessels were inoculated with *A*. *mellea*, five with *D*. *caespitosa*, and two vessels were left as control (non-inoculated). ‘Guardian^®^’ and ‘MP-29’ were co-cultured with both fungi for three weeks with sample points considered at 3, 7, 10 (except ‘MP-29’), 14, and 21 days after plants were transferred to inoculated vessels. ‘14–4’ was co-cultured with both fungi for four weeks, and samples were collected at 3, 7, 14, 21, and 28 days after the co-culture began. Non-inoculated (control) vessels for each genotype were harvested at 0 and the end of the experiment for each genotype. Roots and leaves were separated and weighted at each sample point for each *Prunus* genotype and treatment. Similarly, fungus biomass for those inoculated vessels was scaled for the same sample points.

## 4. Results and discussion

This protocol developed and validated an aseptic *in vitro* co-culture system that supported the simultaneous growth of plants of the genus *Prunus* and the soil-borne fungi *Armillaria mellea* and *Desarmillaria caespitosa*. This approach successfully overcame most of the disadvantages identified in the previously developed *in vitro* co-culture systems, where the limitation in the number of plants available for testing and the long inoculation times were identified as critical [8, 25, 26].

**Fig 2:**
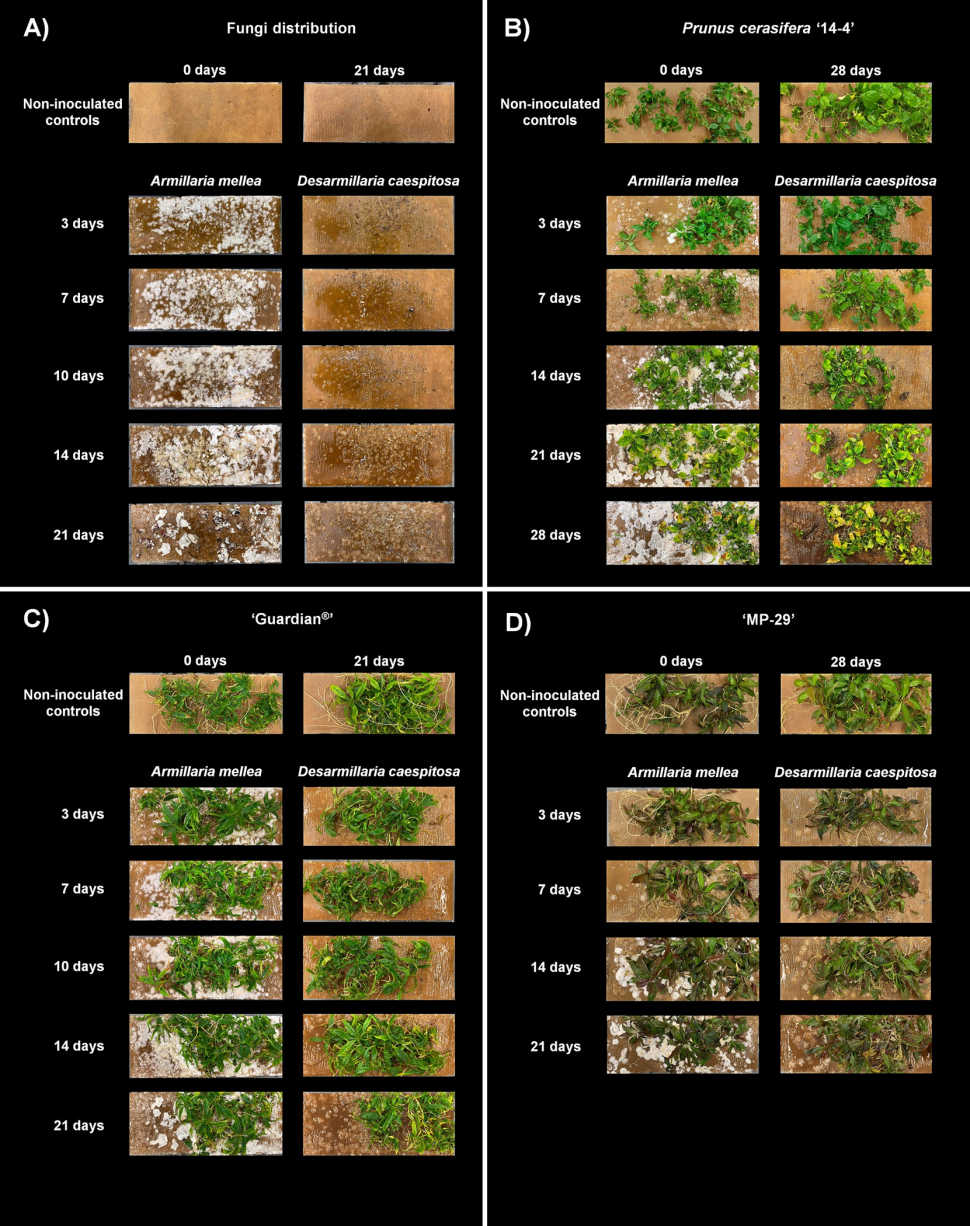
(**A**) *Armillaria mellea* and *Desarmillaria caespitosa* growth in the fiber-supported paper at 3, 7, 10, 14, and 21 days after co-culture establishment. Growth of *Prunus cerasifera* ‘14–4’ (**A**), ‘Guardian^®^’ (**B**), and ‘MP-29’ (**C**) in co-culture with *Armillaria mellea* and *Desarmillaria caespitosa* at different days after the co-culture establishment. Non-inoculated controls at the beginning and end of the co-culture establishment.

**Fig 3:**
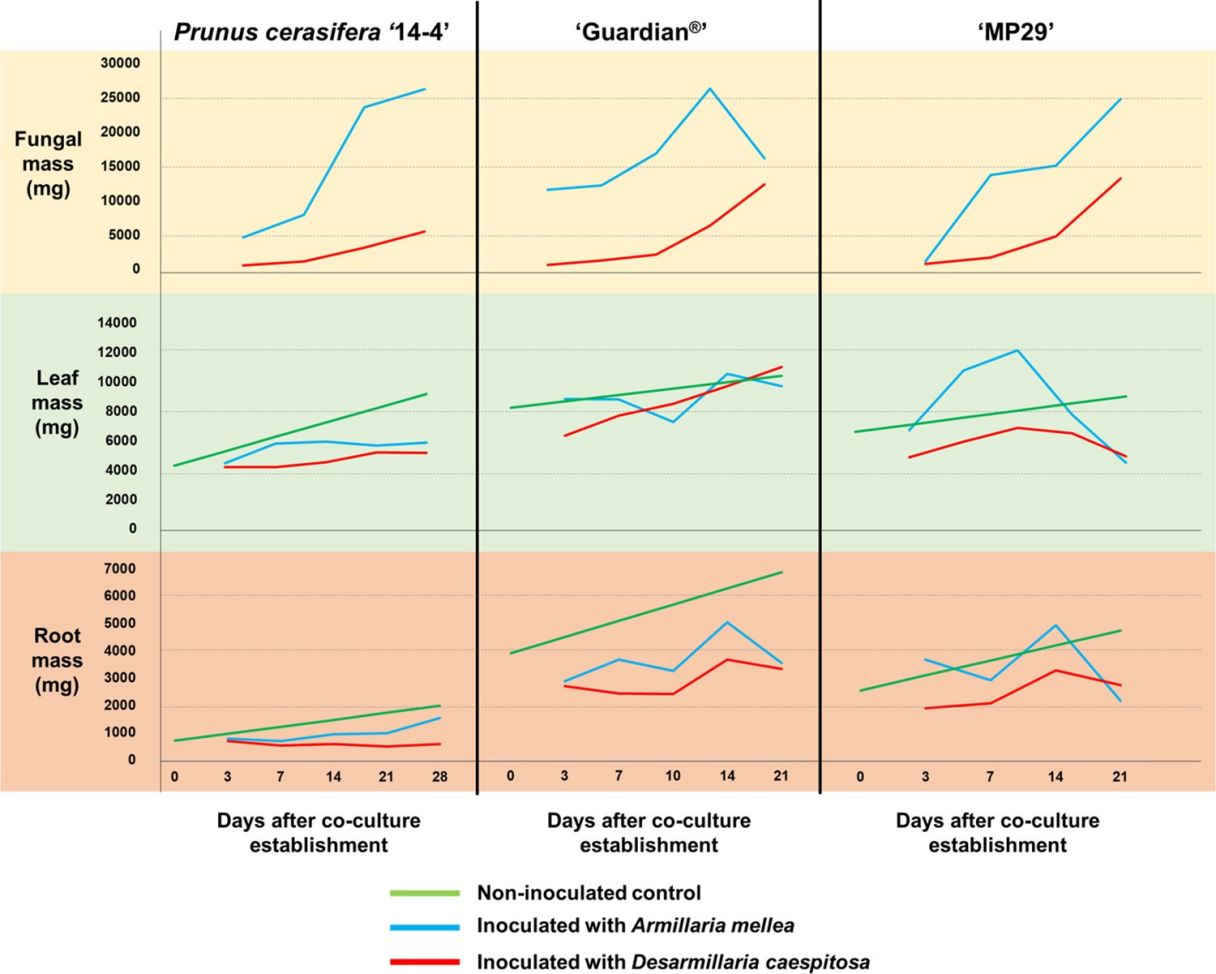
Progression of fungal, leaf, and root mass during the co-culture of *Prunus cerasifera* ‘14–4’, ‘Guardian^®^’, and ‘MP-29’ with *Armillaria mellea* and *Desarmillaria caespitosa*.

During the development and validation of this approach, *A*. *mellea* and *D*. *caespitosa* grew well on the Southern Sun BioSystems vessels (Fig 2). The continuous tilting movement of the vessels created an agitated thin-film slurry with an even distribution of fungi while both organisms had continuous availability of water, nutrients and oxygen. Signs of mycelia growth for both species were first visible on the surface of inoculated vessels five days after inoculation. However, *D*. *caespitosa* showed slower growth than *A*. *mellea* under this *in vitro* approach (Figs 2 and 3). Because of that, after three weeks of co-culture, the amount of mycelium produced by *A*. *mellea* was approximately three times larger than that produced by *D*. *caespitosa* (Figs 2 and 3). Nevertheless, this difference in fungi mass did not influence plant infection, as all roots sampled 21 days after co-culture establishment were covered by the fungi.

Co-culturing *A*. *mellea* and *D*. *caespitosa* with ‘Guardian^®^’, *Prunus cerasifera* ‘14–4’, and ‘MP-29’ plants using the *in vitro* approach was successful (Figs 2 and 3). Fungal infection exhibited substantial efficacy and a large biomass of root tissue was available over several weeks of fungal infection (Fig 3). The rootstocks, *P*. *persica* ‘Guardian^®^’ and the hybrid ‘MP-29’, yielded 2–4 g of root mass per vessel, and the non-domesticated *P*. *cerasifera* yielded 0.5–1.5 g per vessel, which provided adequate quantities for downstream transcriptome or metabolome analyses of the infected root tissues. Inoculation of the vessels before plant transfer ensured that roots were in contact with fungi from the moment plants were transferred to vessels, allowing a fast infection. Symptoms of root infections, indicated by the presence of rotten root tissues, were observed at various times (typically after one week of co-culture) depending on genotype susceptibility to fungi (S2 Fig), indicating early successful inoculations.

Most co-culture systems employed in studying plant-pathogen interactions utilize solid or semisolid growing media, resulting in a significant delay in the onset of infection. This delay arises from the necessity for the physical encounter between fungal mycelia and plant roots before the infection process can begin [8, 9, 25]. Through the implementation of the fiber-supported liquid co-culture approach wherein fungal inoculation precedes plant transfer, the initiation of infection occurs in close temporal proximity to the plant transfer to inoculated vessels. Additionally, higher amounts of biomass obtained in liquid cultures than on agar were reported in some plant species [30], producing higher amounts of tissues and biologically active plant-derived compounds like metabolites. However, it is also important to note that *Prunus* species are not typically grown in water-based media, thus submerging *Prunus* plants in liquid-based mediums could lead to physiological and transcriptomic changes [9, 36]. This limitation should be taken into account when considering co-culture systems, depending on the goal of the experiment.

## 5. Conclusions

This protocol explains the development and validation of an *in vitro* co-culture system strategically designed for the straightforward inoculations of *Prunus* ssp. roots with *Armillaria/Desarmillaria* spp. Despite being designed for ARR resistance evaluation in *Prunus* spp., this approach could be implemented for any disease evaluation caused by many different fungal root-invading pathogens. The fiber-supported paper and the controlled environment minimize the risk of contamination and reduce the temporal and material resources required for field evaluations, thereby enabling the phenotyping of numerous plants. Implementation of this technique promotes sufficient root tissue growth for various downstream analyses and aids in breeding plants for disease resistance.

## Supporting information

S1 File Step-by-step protocol, also available on protocols.io. (PDF)

S1 Fig Plastic rectangular vessel (110 × 297 mm; Southern Sun BioSystems, Hodges, SC, USA) used in the co-culture experiment. (TIF)

S2 Fig Comparison of ‘MP-29’ roots under control (non-inoculated) conditions and after three weeks of co-culture with *Armillaria mellea* and *Desarmillaria caespitosa*. (TIF)

## References

[1] Zewdu G, Tamene W, Assefa Z, Tamiru T, Bankseni L, Borja M, et al. The role of modern plant breeding to control plant disease: a review. Agricultural J. 2022; 17(4): 19–27. 10.1016/j.tifs.2020.03.042

[2] Raabe RD. Testing grape rootstocks for resistance to the oak root fungus. California Plant Pathol 1979; 46: 3–4.

[3] Guillaumin JJ, Pierson J, Grassely C. The susceptibility of different *Prunus* species used as stone fruit rootstocks to *Armillaria mellea* (sensu stricto). Seventh International Conference on Root and Butt Rots. International Union of Forestry Research Organizations, Vernon and Victoria, BC, Canada 1989.

[4] Beckman TG, Pusey PL. Field testing peach rootstocks for resistance to *Armillaria* root rot. HortScience 2001; 36: 101–103. 10.21273/HORTSCI.36.1.101

[5] Hao J, Yang ME, Davis RM. Effect of soil inoculum density of *Fusarium oxysporum* f. sp. *vasinfectum* Race 4 on disease development in cotton. Plant Dis. 2009; 93(12) 1324–1328. 10.1094/PDIS-93-12-1324 30759502

[6] Shishido K, Murakami H, Kanda D, Fuji S, Toda T, Furuya H. Effect of soil inoculum density and temperature on the incidence of Cucumber black root rot. Plant Dis. 2015; 100(1): 5–235 10.1094/PDIS-12-14-1287-RE 30688561

[7] Lebeda A, Švábová L. *In vitro* screening methods for assessing plant disease resistance. In Mass screening techniques for selecting crops resistant to disease. FAO/IAEA. Vienna, 2010. Pp 5–45.

[8] Adelberg J, Naylor-Adelberg J, Miller S, Gasic J, Schnabel G, Bryson P, et al. *In vitro* co-culture system for *Prunus* spp. and *Armillaria mellea* in phenolic foam rooting matric. Plant 2021; 57: 387–397. 10.1007/s11627-020-10136-2

[9] Parris SM, Jeffers SN, Olvey JM, Adelberg JW, Wen L, Udall JA, et al. An in vitro co-culture system for rapid differential response to *Fusarion oxysporum* f. sp vasinfectum Race 4 in three cotton cultivars. Plant Dis 2022; 106(3): 990–995. 10.1094/PDIS-08-21-1743-RE 34705484

[10] Fenning TM. The use of tissue culture and *in vitro* approaches for the study of tree diseases. Plant Cell, Tissue and Organ Culture 2019; 136: 415–430. 10.1007/s11240-018-01531-0

[11] Pérez-Clemente RM, Gómez-Cadenas A. *In vitro* tissue culture, a tool for the study and breeding of plants subjected to abiotic stress conditions. *In* Recent Advances in Plant *in vitro* Culture 2012; 91–108. 10.5772/50671

[12] Devkota P, Hammerschmidt R. The infection process of *Armillaria mellea* and *Armillaria solidides*. Phys. and Mol. Plant Path. 2020; 112: 101543. 10.1016/j.pmpp.2020.101543

[13] Hood IA, Redfern DB, Kile GA. Armillaria in planted hosts. In Armillaria root disease, USDA Forest Service, pp. 1991; 122–149.

[14] Baumgartner K. Root collar excavation for postinfection control of *Armillaria* root disease of grapevine. Plant Dis. 2004; 88: 1235–1240. 10.1094/PDIS.2004.88.11.1235 30795318

[15] Schnabel G, Ash JS, Bryson PK. Identification and characterization of *Armillaria* tabescens from the southeastern United States. Mycol. Res. 2005; 109: 1208–1222. 10.1017/s0953756205003916 16279414

[16] Herrin G. An economic impact and investment analysis of Armillaria root rot in the United States peach industry. All Theses 2022; 3949. https://tigerprints.clemson.edu/all_theses/3949

[17] Beckman TG, Okie WR, Nyczepir AP, Pusey PL, Reilly CC. Relative susceptibility of peach and plum germplasm to *Armillaria* root rot. HortScience 1998; 33: 1062–1065. 10.21273/HORTSCI.33.6.1062

[18] Beckman TG, Chaparro JX, Sherman WB. ‘MP-29’, a clonal interspecific hybrid rootstock for peach. HortScience 2012; 47:128–131. 10.21273/HORTSCI.47.1.128

[19] Schnabel G, Agudelo P, Henderson GW, Rollins PA. Aboveground root collar excavation of peach trees for *Armillaria* root rot management. Plant Dis. 2012; 96: 681–686. 10.1094/PDIS-06-11-0493 30727526

[20] Miller SB, Gasic K, Reighard G, Henderson WG, Rollins PA, Vassalos, M. et al. Preventative root-collar excavation reduces peach tree mortality caused by Armillaria root rot on replant sites. Plant Dis. 2020; 104(5): 1274–1279. doi: 10.1094/PDIS-09-19-1831-RE 32213125

[21] Gasic K, Adelberg J, Baumgartner K, Brannen PM, Cai L, Calle A, et al. Solutions to the *Armillaria* root rot affecting the US stone fruit industry. Acta Hortic. 2022; 1352, 501–508 10.17660/ActaHortic.2022.1352.68

[22] Thomas HE, Roberts C, Amstutz A. Rootstock susceptibility to *Armillaria mellea*. Phytopathology 1948; 38:152–154.

[23] Mansilla JP, Aguin O, Sainz MJ. A fast method for production of *Armillaria* inoculum. Mycologia 2001; 93: 612–615. 10.1080/00275514.2001.12063191

[24] Raziq F, Fox RTV. Combinations of fungal antagonists for biological control of *Armillaria* root rot of strawberry plants. Biol Agr Hort 2005; 23: 45–57. 10.1080/01448765.2005.9755307

[25] Baumgartner K, Fujiyoshi P, Ledbetter C, Duncan R, Kluepfel D. Screening almond rootstocks for sources of resistance to *Armillaria* root disease. HortScience 2018; 53: 4–8. 10.21273/HORTSCI12038-17

[26] Devkota P, Hammerschmidt R. A rapid and holistic approach to screen susceptibility of *Prunus* species to *Armillaria* root rot. Forest Pathol 2019; 49. 10.1111/efp.12547

[27] Devkota P, Iezzoni A, Gasic K, Reighard G, Hammerschmidt R. Evaluation of susceptibility of *Prunus* rootstock genotypes to *Armillaria* and *Desarmillaria* species. Eur. J. Plant Pathol. 2020; 158: 177–193. 10.1007/s10658-020-02065-y

[28] Economou AS. From microcutting to microplant establishment: key points to consider for maximum success in woody plants. Acta Hort 2013; 988: 43–56. 10.17660/ActaHortic.2013.988.3

[29] Cai L, Adelberg J, Naylor-Adelberg J, Schnabel G, Calle A, Li Z, et al. Transcriptomic reveal the genetic coordination of early defense to Armillaria root rot (ARR) in *Prunus* spp. Front. in Plant Sci. 2023; 14: 1181153. 37332708 10.3389/fpls.2023.1181153 PMC10274510

[30] Tascan A, Adelberg JW, Joshee N, Yadav AK, Tascan M. Liquid culture system for Scutellaria species. Act Hort 2007; 756: 163–170. 10.17660/ActaHortic.2007.756.18

[31] Tascan A, Adelberg JW, Tascan M, Rimando A, Joshee N, Yadav A. Hyperhydricity and flavonoid content of Scutellaria species in vitro on polyester-supported liquid culture systems. HortScience 2010; 45(11): 1723–1728. https://doir.org/10.21273/HORTSCI.45.11.1723

[32] Murashige T, Skoog F. A revised medium for rapid growth and bio assays with tobacco tissue cultures. Physiologia Plantarum 1962; 15(3): 473–497. 10.1111/j.1399-3054.1962.tb08052.x

[33] Lloyd G, McCown B (1981) Commercially-feasible micropropagation of mountain laurel, *Kalmia latifolia*, by use of shoot-tip culture. Comb Proc Intl Plant Prop Soc 2010; 30: 421–427.

[34] Kalinina A, Brown D (2007) Micropropagation of ornamental *Prunus* spp. GF305 peach, a Prunus viral indicator. Plant Cell Reports 26: 927–935. 10.1007/s00299-007-0315-x 17323085

[35] Adelberg J, Simpson EP. Intermittent immersion vessel apparatus and process for plant propagation. US Patent 2004; 6: 753, 178

[36] Adelberg J, Naylor-Adelberg J, Rapaka V. A novel rooting matrix and vessel system resulted in larger plants and faster growth during greenhouse acclimatation of *Hydrangea quercifolia* ‘Sikes dwarf’. Propagation of Ornamental Plants 2015; 15(3): 89–94.

