# Supplementary material for: In vitro co-culture system for investigating Armillaria root rot in Prunus spp. using a fiber-supported liquid approach
Source: PLoS One. 2024 Sep 23;19(9):e0310314. doi: 10.1371/journal.pone.0310314 (PMC11419352; doi:10.1371/journal.pone.0310314)
Supplement: S1 File — (PDF) [file pone.0310314.s001.pdf]

Jul 03, 2024

In vitro co-culture system using a fiber-supported liquid approach

DOI
dx.doi.org/10.17504/protocols.io.rm7vzj56xlx1/v1

Alejandro Calle[1], Jeffrey Adelberg[2], Guido Schnabel[2], Jacqueline Naylor-Adelberg[2], Jhulia Gelain[2], Yeter Karakoc[2], Jared Weaver[2], Christopher Saski[2], Ksenija Gasic[2]

[1]Fruit Production Program, Institut de Recerca i Tecnologia Agroalimentàries (IRTA);
[2]Department of Plant and Environmental Sciences. Clemson University. Clemson, SC, USA

Ksenija Gasic
Clemson University

OPEN ACCESS

DOI: dx.doi.org/10.17504/protocols.io.rm7vzj56xlx1/v1

Protocol Citation: Alejandro Calle, Jeffrey Adelberg, Guido Schnabel, Jacqueline Naylor-Adelberg, Jhulia Gelain, Yeter Karakoc, Jared Weaver, Christopher Saski, Ksenija Gasic 2024. In vitro co-culture system using a fiber-supported liquid approach. **protocols.io** https://dx.doi.org/10.17504/protocols.io.rm7vzj56xlx1/v1

License: This is an open access protocol distributed under the terms of the **Creative Commons Attribution License**, which permits unrestricted use, distribution, and reproduction in any medium, provided the original author and source are credited

Protocol status: Working
We use this protocol and it's working

Created: July 02, 2024

Last Modified: July 03, 2024

Protocol Integer ID: 102750

Keywords: Phenotyping, plant disease infection, fungal inoculation, plant culture

Funders Acknowledgement:
USDA-NIFA
Grant ID: 2020-51181-32142.

Abstract

In vitro co-culture techniques that allow the growth of plants and pathogens under controlled environmental conditions are being used to re-create host plant infection. These approaches reduce infection times, promote reproducibility, and enable a rapid evaluation of plant-pathogen interactions. As a result, these systems have become essential in breeding programs aimed at developing plant resistance to diseases. In this study, we developed and validated an *in vitro* co-culture system to investigate the Armillaria root rot (ARR) affecting *Prunus* spp. This disease, caused by fungi *Armillaria* spp. and *Desarmillaria caespitosa*, poses
a severe threat to the stone and nut fruit industry due to the susceptibility of most commercial rootstocks to infection and the lack of effective management options for its control. The system consists of a fiber-supported liquid approach in sterile plastic vessels that allows a fast and reproducible fungal infection under controlled environmental conditions. The floor of the vessels was covered with a polyester-fiber matte and a germination paper that served as an interface between the mycelia and the plant roots. The vessels were subjected to inoculation with *Armillaria mellea* and *D. caespitosa*, and three *Prunus* genotypes ('Guardian®', 'MP-29', and *Prunus cerasifera* '14-4') were co-cultured with both fungi. Disease progression and plant and fungal biomass were monitored during co-culture. The presented *in vitro* co-culture approach facilitates the concurrent growth of *Armillaria/Desarmillaria* spp. and *Prunus* spp., excluding most of the limitations associated with greenhouses and field experiments. This system provides consistent and reproducible conditions for investigating a prominent plant disease affecting *Prunus* spp.

Materials

Materials

- 20 × 150 mm culture tubes (Stellar Scientific; Baltimore, MD, USA, Cat. Nº:
SKU:GS-1522)
- Autoclavable polypropylene culture tube closures (General Laboratory Supply; Pasadena, TX, USA, Cat. Nº: T3054-4)
- Magenta™ GA-7 vessels (Merck, Darmstadt, Germany, Cat. Nº: V8505-25EA)
- Petri plates (VWR International, Radnor, PA, USA, Cat. Nº: 391-0579)
- Parafilm 'M' laboratory film (Sigma Aldrich; Darmstadt, Germany; Cat. Nº: P7668)
- 15 mL Pyrex® Ten Broeck tissue grinder with a pour spout (Corning, Tewksbury, MA, USA; Cat. Nº: 7727-15)
- Ultra-clear porous cellophane sheet (0.1 mm thick) (Research Products International, Mount Prospect, IL, USA; Cat. Nº: 1080)
- Lazy-L spreader (Merck, Darmstadt, Germany, Cat. Nº: Z376779)
- 1.5 mL Eppendorf® tubes (Thermo Fisher Scientific Inc, Waltham, WA, USA; Cat. Nº: 0030120175)
- Rectangular vessels (110 × 297 mm; Southern Sun BioSystems, Hodges, SC, USA)
- Polyester fiber matte (BioStrate™ Felt; Cropking Inc., Lodi, OH, USA)
- Germination paper (Anchor Paper Co., St. Paul, MN, USA)
- Polyvinyl chloride (PVC) sealing film (Phytotech Laboratories, Shawnee Mission, KS, USA; Cat. Nº: A003)

Equipment

- Laminar flow hood
- Autoclave
- Articulated rocker arm
- Fungal growth incubator
- LED light NutriLED, Hubbell Lighting, Greenville, SC, USA)

Establishment of Plant Cultures

1 *Establishment of plant cultures from dormant shoots*

1.1 Collect dormant shoots, cut them (3 cm in length),and cleanse them by submerging in 70% ethanol for 1 minute, followed by rinsing with sterile deionized water. Then, immerse the shoots in a 10% bleach solution for 10 minutes, and rinse them twice with deionized water (Figure 1).

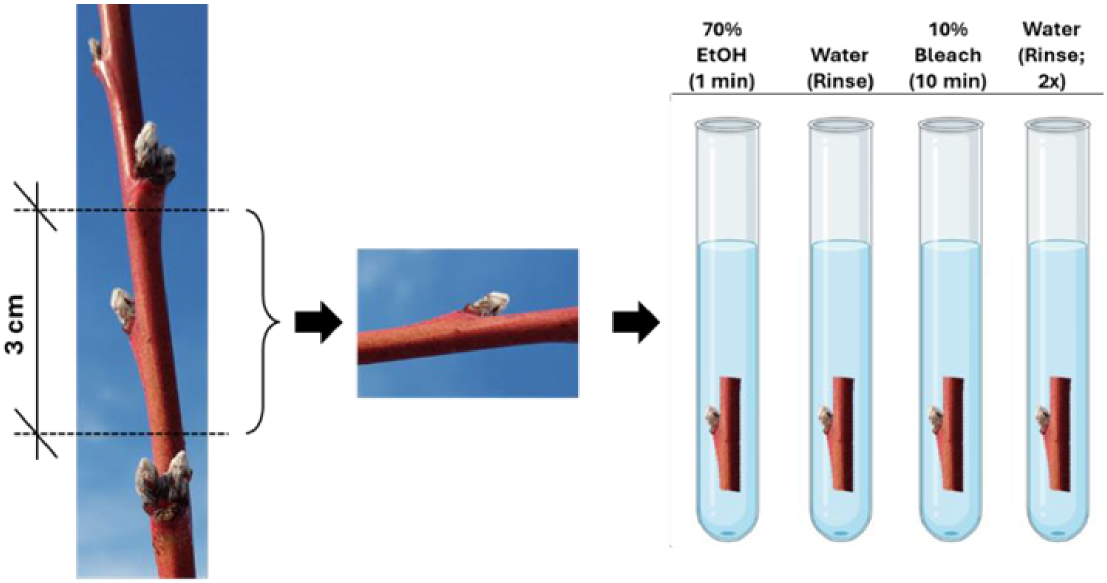

Figure 1. Cleaning process of dormant shoots

1.2 Peel the vegetative shoot buds and transfer them into culture tubes containing 20 mL of Murashige and Skoog agar media. Place the shoot vertically ensuring that the bud is 1 cm above the agar media.

2 *Establishment of plant cultures from seeds*

2.1 Clean fruit exocarps with 20% bleach for 10 minutes, followed by a 10-minute immersion in 70% ethanol.

2.2 Extract seeds within a laminar flow hood and transfer them aseptically into culture tubes containing Woody Plant Medium. Allow them to undergo stratification for ten weeks in darkness at 4 °C.

2.3 Upon germination (Figure 2), micropropagate shoot tips in culture vessels(Magenta GA-7) using an agar-based medium

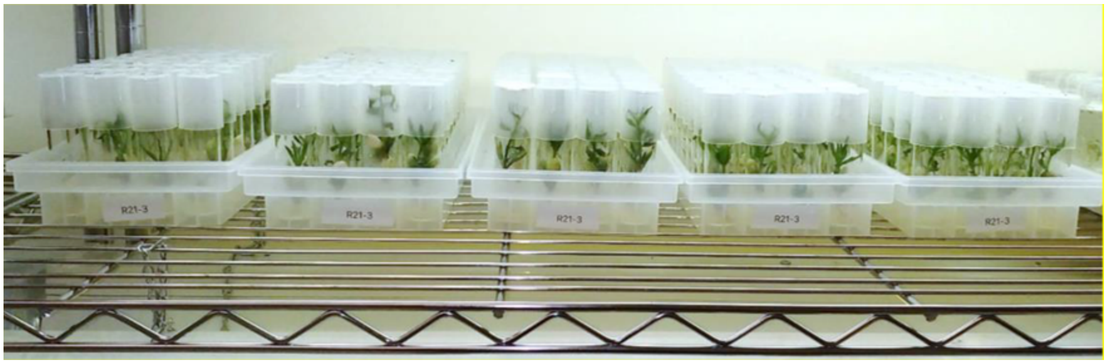

Figure 2. Germinated seeds in tubes.

3 *Maintenance of stock plants*

3.1 Sustain stock plants in Magenta GA-7 vessels by transferring shoot tips every five weeks onto a fresh medium.

3.2 Maintain vessels under a photosynthetic photon flux density of 20 µmol/s/m2, with a 16-hour photoperiod at 24°C.
Optional: When the When the presence of hyper multiplication, an occasional resting cycle with 16 µM indole-3-acetic acid (resting media) is recommended.

3.3

Fungi Preservation

4 Propagate fungal cultures in Petri plates by placing two plugs (0.5 × 0.5 cm) from the youngest part of the colony (Figure 3).

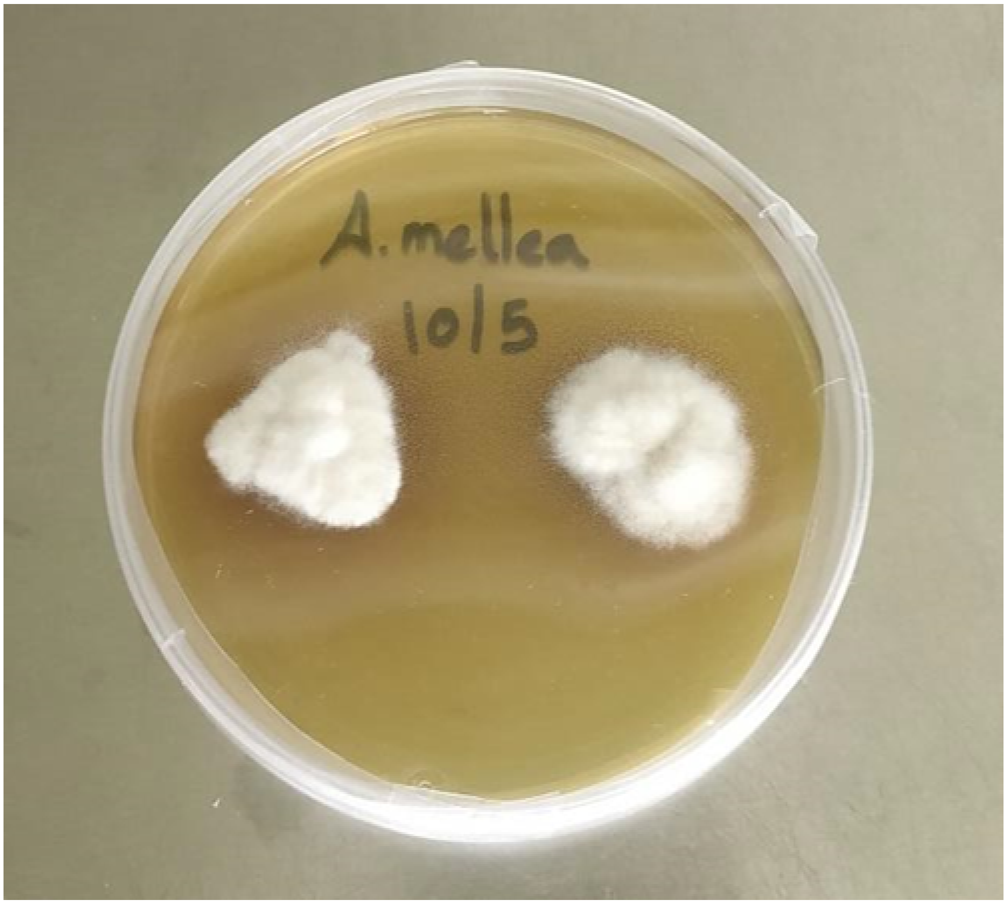

Figure 3. One-week-old *Armillaria mellea* cultures

5 Seal plates with parafilm and maintain in the dark at 20 °C.

6 Refresh every 14 days by transferring mycelial plugs to fresh MEA plates to ensure active fungal growth.

Inoculum preparation

7 Extract three ten-millimeter-diameter plugs from the edge of two-week-old colonies

8 Remove most of the agar plug and homogenize mycelium with 5 mL of sterile water using a sterilized 15 mL tissue grinder (Figure 4).

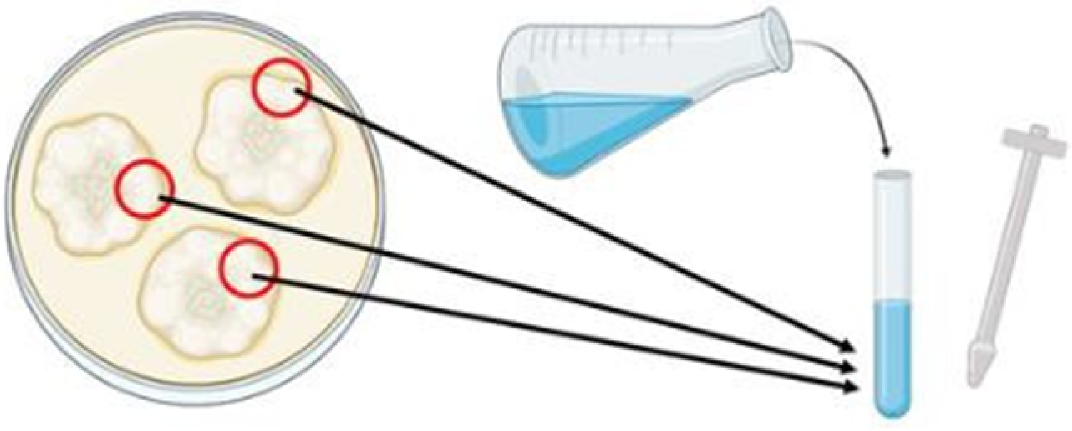

Figure 4. Fungi homogenization in sterile water

9 Place a sterile ultra-clear porous cellophane sheet on top of a Petri dish containing malt extract agar media and pour 600 µL of homogenate

10 Spread homogenate uniformly over the entire plate using a cell spreader and incubate the plate in the dark for 14 days.

11 Prepare the mycelium suspension for inoculum by taking a 2 × 2 cm plug from the previously prepared
plate and homogenate with 7 mL of sterile water using a tissue grinder, and aliquoted in 1.5 mL Eppendorf tubes (Figure 5).

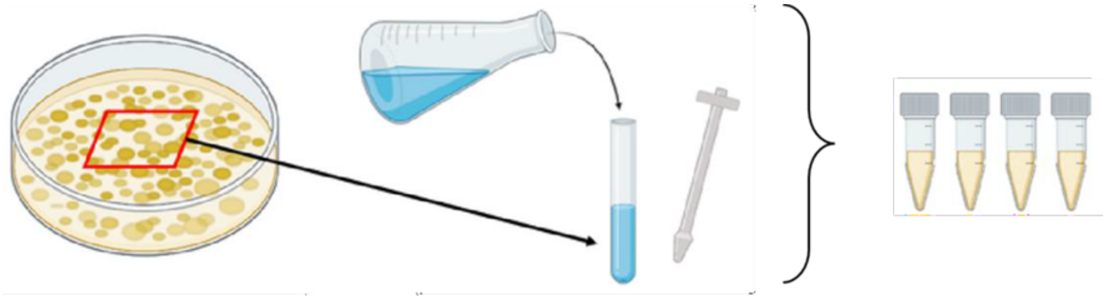

Figure 5. Preparation of the inoculum suspension for infection.

Co-culture (plant-fungi) establishment

12 Autoclave (121 °C for 20 min) rectangular plastic vessels (110 × 297 mm; Southern Sun BioSystems) and after cooling down to room temperature, set the fiber-supported paper on the floor of each vessel (Figure 6).

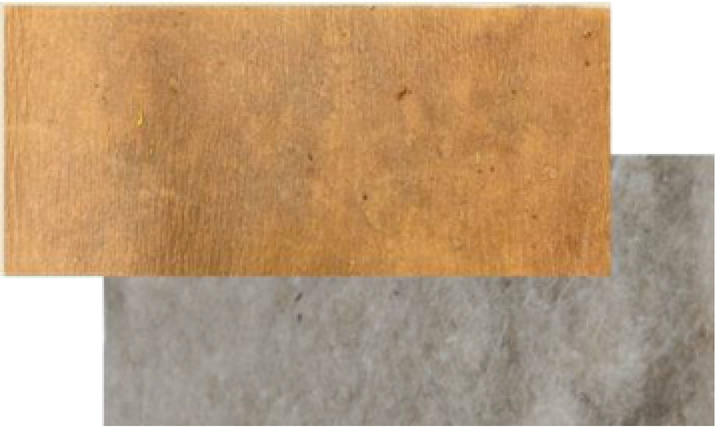

Figure 6. a) Fiber-supported paper.

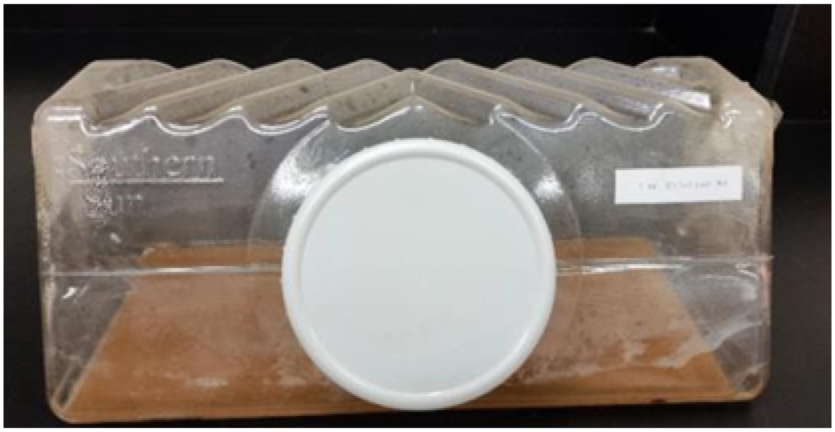

Figure 6. b) Southern Sun BioSystems with fiber-supported paper inside.

13 Add 175 mL of plant growth regulator-free liquid ‘New *Prunus* Medium’ to each vessel.

14 Transfer fifteen *in vitro* plants from an agar-based medium to each vessel removing the agar and sealing vessels with polyvinyl chloride film (Figure 7).

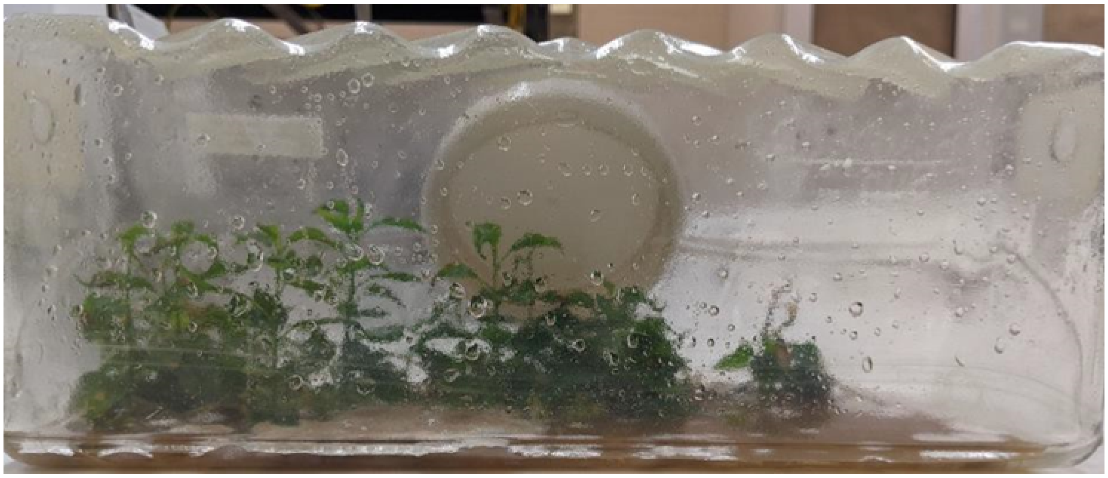

Figure 7. *In vitro* plants growing in the Southern Sun BioSystems vessels.

15 Place vessels on a rocker's arm with an articulated shelf that providesone swing every 15 min.

16 Use another set of rectangular Southern Sun BioSystems vessels containing 175 mL of 'New *Prunus* Medium', fiber matte, and germination paper and add 1 mL of the mycelium suspension for inoculum.

17 Seal the vessels with PVC film and place them on an automatic rocker arm at 5 rpm under µM/m2/s LED light 2 red 1 blue and 16 h/day photoperiod at 24 ºC.

18 After ten and seventeen days of inoculation with *A. mellea* and *D. caespitosa*, respectively, transfer the *in vitro* rooted plants from the liquid media to the inoculated vessels.

19 Add 60 mL of 'New *Prunus* Medium' without any plant growth regulator just before plant transferring.

20 Seal the vessels with PVC film and place them on an automatic rocker arm at 5 rpm under µM/m2/s LED light 2 red 1 blue and 16 h/day photoperiod at 24 ºC (Figure 8).

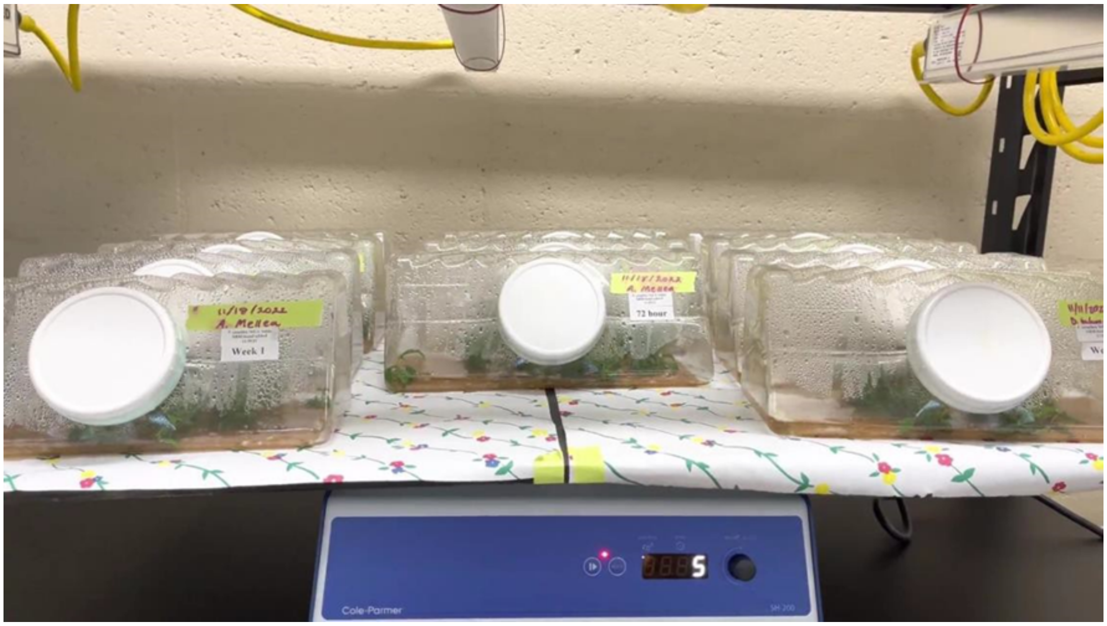

Figure 8. Southern Sun BioSystems vessels on rocking platform with an articulated shelf (5rpm) exposed to 20 µmol/s/m², 16h/day at 24 °C.

21 Collect tissues when needed.

Protocol references

Adelberg J, Simpson EP. Intermittent immersion vessel apparatus and process for plant propagation. US Patent 2004; 6: 753, 178

Adelberg J, Naylor-Adelberg J, Miller S, Gasic J, Schnabel G, Bryson P, et al. *In vitro* co-culture system for *Prunus* spp. and *Armillaria mellea* in phenolic foam rooting matric. Plant 2021; 57: 387-397. https://doi.org/10.1007/s11627-020-10136-2

Adelberg J, Naylor-Adelberg J, Rapaka V. A novel rooting matrix and vessel system resulted in larger plants and faster growth during greenhouse acclimatation of *Hydrangea quercifolia* 'Sikes dwarf'. Propagation of Ornamental Plants 2015; 15(3): 89-94.

Baumgartner K. Root collar excavation for postinfection control of *Armillaria* root disease of grapevine. Plant Dis. 2004; 88: 1235–1240. https://doi.org/**10.1094/PDIS.2004.88.11.1235**

Baumgartner K, Fujiyoshi P, Ledbetter C, Duncan R, Kluepfel D. Screening almond rootstocks for sources of resistance to *Armillaria* root disease. HortScience 2018; 53: 4–8. https://doi.org/10.21273/HORTSCI12038-17

Beckman TG, Okie WR, Nyczepir AP, Pusey PL, Reilly CC. Relative susceptibility of peach and plum germplasm to *Armillaria* root rot. HortScience 1998; 33: 1062–1065. https://doi.org/10.21273/HORTSCI.33.6.1062

Beckman TG, Pusey PL. Field testing peach rootstocks for resistance to *Armillaria* root rot. HortScience 2001; 36: 101–103. https://doi.org/10.21273/HORTSCI.36.1.101

Beckman TG, Chaparro JX, Sherman WB. 'MP-29', a clonal interspecific hybrid rootstock for peach. HortScience 2012; 47:128–131. https://doi.org/10.21273/HORTSCI.47.1.128

Cai L, Adelberg J, Naylor-Adelberg J, Schnabel G, Calle A, Li Z, et al. Transcriptomic reveal the genetic coordination of early defense to Armillaria root rot (ARR) in *Prunus* spp. Front. in Plant Sci. 2023; 14: 1181153.

Devkota P, Hammerschmidt R. A rapid and holistic approach to screen susceptibility of *Prunus* species to *Armillaria* root rot. Forest Pathol 2019; 49. https://doi.org/10.1111/efp.12547

Devkota P, Hammerschmidt R. The infection process of *Armillaria mellea* and *Armillaria solidides*. Phys. and Mol. Plant Path. 2020; 112: 101543. https://doi.org/10.1016/j.pmpp.2020.101543

Devkota P, Iezzoni A, Gasic K, Reighard G, Hammerschmidt R. Evaluation of susceptibility of *Prunus* rootstock genotypes to *Armillaria* and *Desarmillaria* species. Eur. J. Plant Pathol. 2020; 158: 177-193. https://doi.org/10.1007/s10658-020-02065-y

Economou AS. From microcutting to microplant establishment: key points to consider for maximum success in woody plants. Acta Hort 2013; 988: 43–56. https://doi.org/10.17660/ActaHortic.2013.988.3

Fenning TM. The use of tissue culture and *in vitro* approaches for the study of tree diseases. Plant Cell, Tissue and Organ Culture 2019; 136: 415-430. https://doi.org/10.1007/s11240-018-01531-0

Gasic K, Adelberg J, Baumgartner K, Brannen PM, Cai L, Calle A, et al. Solutions to the *Armillaria* root rot affecting the US stone fruit industry. Acta Hortic. 2022; 1352, 501-508 https://doi.org/10.17660/ActaHortic.2022.1352.68

Guillaumin JJ, Pierson J, Grassely C. The susceptibility of different *Prunus* species used as stone fruit rootstocks to *Armillaria mellea* (sensu stricto). Seventh International Conference on Root and Butt Rots. International Union of Forestry Research Organizations, Vernon and Victoria, BC, Canada 1989.

Hao J, Yang ME, Davis RM. Effect of soil inoculum density of *Fusarium oxysporum* f. sp. *vasinfectum* Race 4 on disease development in cotton. Plant Dis. 2009; 93(12) 1324-1328. https://doi.org/**10.1094/PDIS-93-12-1324**

Herrin G. An economic impact and investment analysis of Armillaria root rot in the United States peach industry. All Theses 2022; 3949. https://tigerprints.clemson.edu/all_theses/3949

Hood IA, Redfern DB, Kile GA. Armillaria in planted hosts. In Armillaria root disease, USDA Forest Service, pp. 1991; 122-149.
Lebeda A, Švábová L. *In vitro* screening methods for assessing plant disease resistance. In Mass screening techniques for selecting crops resistant to disease. FAO/IAEA. Vienna, 2010. Pp 5-45.
Lloyd G, McCown B (1981) Commercially-feasible micropropagation of mountain laurel, *Kalmia latifolia*, by use of shoot-tip culture. Comb Proc Intl Plant Prop Soc 2010; 30: 421-427.
Mansilla JP, Aguin O, Sainz MJ. A fast method for production of *Armillaria* inoculum. Mycologia 2001; 93: 612–615. https://doi.org/10.1080/00275514.2001.12063191
Miller SB, Gasic K, Reighard G, Henderson WG, Rollins PA, Vassalos, M. et al. Preventative root-collar excavation reduces peach tree mortality caused by Armillaria root rot on replant sites. Plant Dis. 2020; 104(5): 1274–1279. https://doi.org/10.1094/PDIS-09-19-1831-RE
Murashige T, Skoog F. A revised medium for rapid growth and bio assays with tobacco tissue cultures. Physiologia Plantarum 1962; 15(3): 473-497. https://doi.org/10.1111/j.1399-3054.1962.tb08052.x
Parris SM, Jeffers SN, Olvey JM, Adelberg JW, Wen L, Udall JA, et al. An in vitro co-culture system for rapid differential response to *Fusarion oxysporum* f. sp vasinfectum Race 4 in three cotton cultivars. Plant Dis 2022; 106(3):990-995. https://doi.org/10.1094/PDIS-08-21-1743-RE
Pérez-Clemente RM, Gómez-Cadenas A. *In vitro* tissue culture, a tool for the study and breeding of plants subjected to abiotic stress conditions. *In* Recent Advances in Plant *in vitro* Culture 2012; 91-108. https:// doi.org/**10.5772/50671**
Raabe RD. Testing grape rootstocks for resistance to the oak root fungus. California Plant Pathol 1979; 46: 3-4.
Raziq F, Fox RTV. Combinations of fungal antagonists for biological control of *Armillaria* root rot of strawberry plants. Biol Agr Hort 2005; 23: 45–57. https://doi.org/10.1080/01448765.2005.9755307
Schnabel G, Agudelo P, Henderson GW, Rollins PA. Aboveground root collar excavation of peach trees for *Armillaria* root rot management. Plant Dis. 2012; 96: 681–686. https://doi.org/**10.1094/PDIS-06-11-0493**
Schnabel G, Ash JS, Bryson PK. Identification and characterization of *Armillaria* tabescens from the southeastern United States. Mycol. Res. 2005; 109: 1208–1222. https://doi.org/**10.1017/s0953756205003916**
Shishido K, Murakami H, Kanda D, Fuji S, Toda T, Furuya H. Effect of soil inoculum density and temperature on the incidence of Cucumber black root rot. Plant Dis. 2015; 100(1): 5-235 https://doi.org/10.1094/PDIS-12-14-1287-RE
Tascan A, Adelberg JW, Tascan M, Rimando A, Joshee N, Yadav A. Hyperhydricity and flavonoid content of Scutellaria species in vitro on polyester-supported liquid culture systems. HortScience 2010; 45(11): 1723-1728. https://doir.org/10.21273/HORTSCI.45.11.1723
Tascan A, Adelberg JW, Joshee N, Yadav AK, Tascan M. Liquid culture system for Scutellaria species. Act Hort 2007; 756: 163-170. https://doi.org/10.17660/ActaHortic.2007.756.18
Thomas HE, Roberts C, Amstutz A. Rootstock susceptibility to *Armillaria mellea*. Phytopathology 1948; 38:152–154.
Zewdu G, Tamene W, Assefa Z, Tamiru T, Bankseni L, Borja M, et al. The role of modern plant breeding to control plant disease: a review. Agricultural J. 2022; 17(4): 19-27. https://doi.org/10.1016/j.tifs.2020.03.042
