# Supplementary figures and images for: In vitro co-culture system for investigating Armillaria root rot in Prunus spp. using a fiber-supported liquid approach
Source: PLoS One. 2024 Sep 23;19(9):e0310314. doi: 10.1371/journal.pone.0310314 (PMC11419352; doi:10.1371/journal.pone.0310314)

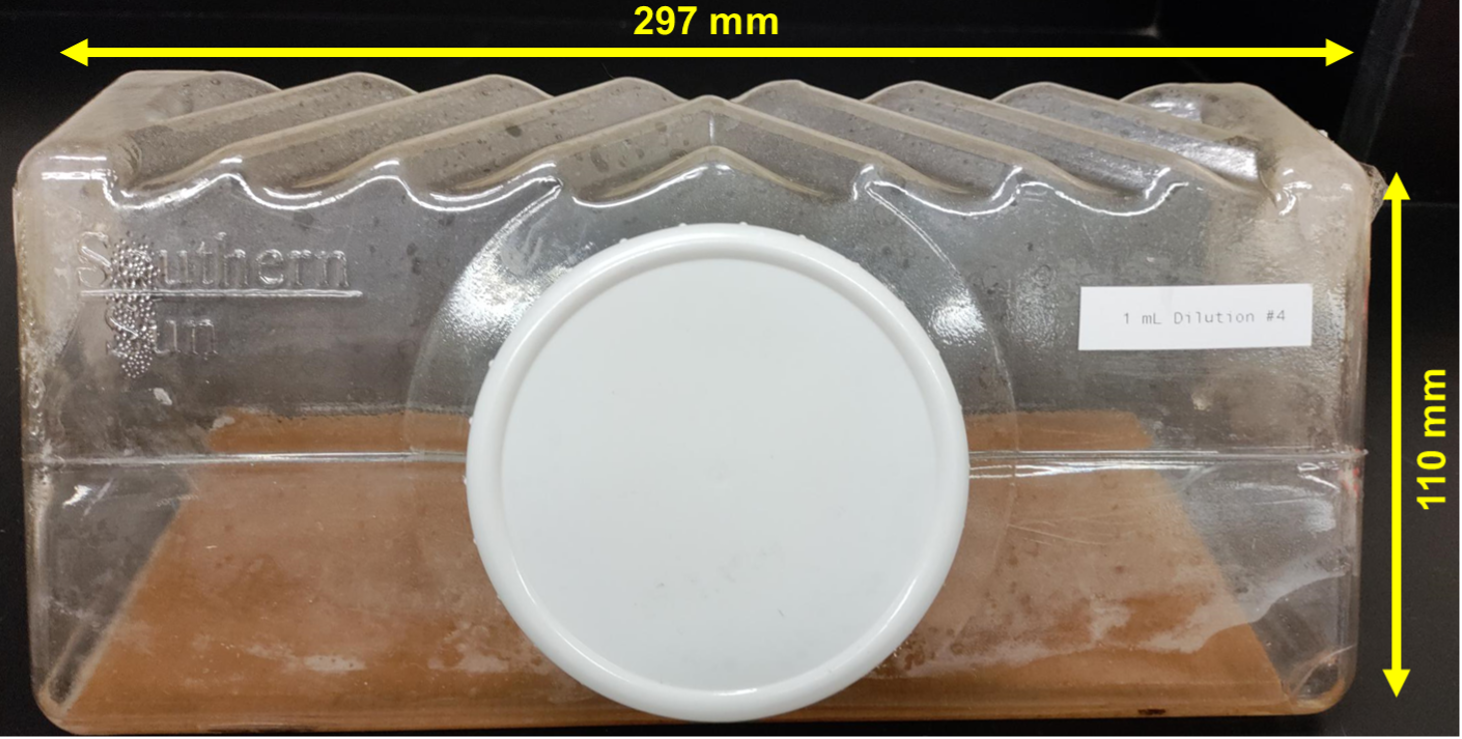

Supplement: S1 Fig — (TIF) [file pone.0310314.s002.tif]

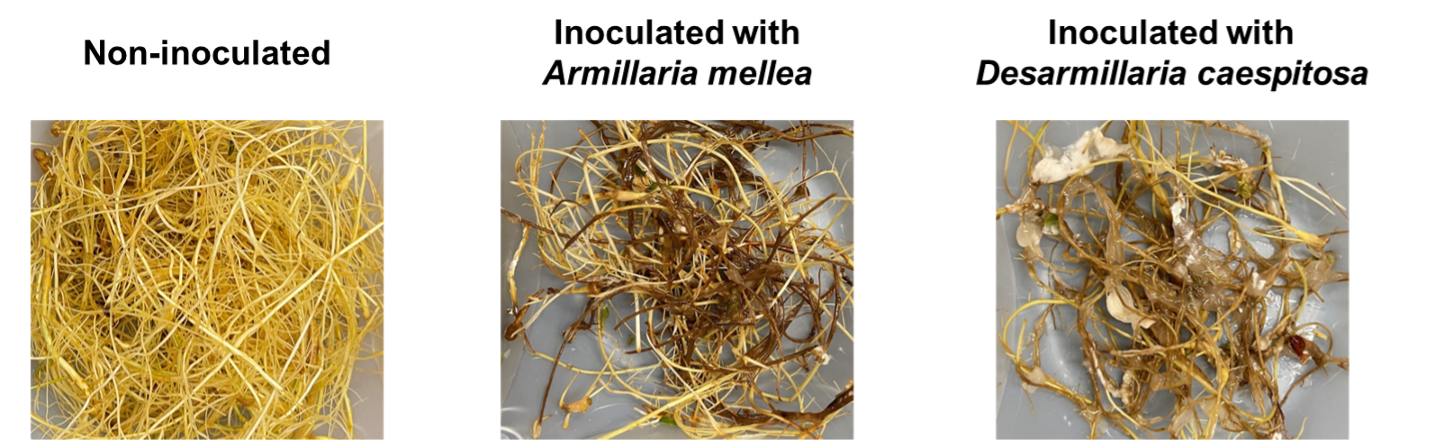

Supplement: S2 Fig — (TIF) [file pone.0310314.s003.tif]
